# LY3009120, a panRAF inhibitor, has significant anti-tumor activity in BRAF and KRAS mutant preclinical models of colorectal cancer

**DOI:** 10.18632/oncotarget.14002

**Published:** 2016-12-16

**Authors:** Eliza Vakana, Susan Pratt, Wayne Blosser, Michele Dowless, Nicholas Simpson, Xiu-Juan Yuan, Susan Jaken, Jason Manro, Jennifer Stephens, Youyan Zhang, Lysiane Huber, Sheng-Bin Peng, Louis F. Stancato

**Affiliations:** ^1^ Oncology Discovery Research, Eli Lilly and Company, Lilly Corporate Center, Indianapolis, IN 46285, USA; ^2^ Discovery Research, Advanced Testing Laboratory, Cincinnati, OH 45242, USA; ^3^ Cell Technologies, Eli Lilly and Company, Lilly Corporate Center, Indianapolis, IN 46285, USA; ^4^ Discovery Statistics, Eli Lilly and Company, Lilly Corporate Center, Indianapolis, IN 46285, USA

**Keywords:** RAS, RAF, signaling pathways, colorectal cancer, xenograft models

## Abstract

Activating mutations in the *KRAS* and *BRAF* genes, leading to hyperactivation of the RAS/RAF/MAPK oncogenic signaling cascade, are common in patients with colorectal cancer (CRC). While selective BRAF inhibitors are efficacious in *BRAF^mut^* melanoma, they have limited efficacy in *BRAF^mut^* CRC patients. In a *RAS^mut^* background, selective BRAF inhibitors are contraindicated due to paradoxical activation of the MAPK pathway through potentiation of CRAF kinase activity. A way to overcome such paradoxical activation is through concurrent inhibition of the kinase activity of both RAF isoforms. Here, we further examined the effects of LY3009120, a panRAF and RAF dimer inhibitor, in human models of CRC with various mutational backgrounds. We demonstrate that LY3009120 induced anti-proliferative effects in *BRAF^mut^* and *KRAS^mut^* CRC cell lines through G1-cell cycle arrest. The anti-proliferative effects of LY3009120 in *KRAS^mut^* CRC cell lines phenocopied molecular inhibition of RAF isoforms by simultaneous siRNA-mediated knockdown of *ARAF*, *BRAF* and *CRAF*. Additionally, LY3009120 displayed significant activity in *in vivo BRAF^mut^* and *KRAS^mut^* CRC xenograft models. Examination of potential resistance to LY3009120 demonstrated RAF-independent ERK and AKT activation in the *KRAS^mut^* CRC cell line HCT 116. These findings describe the preclinical activity of a panRAF inhibitor in a *BRAF^mut^* and *KRAS^mut^* CRC setting.

## INTRODUCTION

In 2014, about 135,000 new cases of colorectal cancer (CRC) were diagnosed in the U.S. alone, along with 50,000 estimated deaths attributed to this disease (NCI statistics). Various genetic and signaling factors contribute to the progression of colon cancer, including microsatellite instability (MSI), mutational inactivation of tumor-suppressor genes such as *APC*, *TP53* and *TGF-*β, as well as aberrant activation of pro-survival pathways such as the RAS/RAF/MEK/ERK and PI3K/mTOR pathways [1].

Activating mutations in the *RAS* and *BRAF* genes occur in 50% and ∼5-10% of patients with CRC, respectively [2] and have been associated with decreased overall survival compared to cases of *KRAS*^WT^ and *BRAF*^WT^ CRC [3]. These mutations result in the constitutive activation of the RAF/MEK/ERK oncogenic signaling cascade, a critical regulator of proliferation and survival [2]. The somatic mutations commonly occur at positions G12 or G13 of *KRAS* and V600 of *BRAF*. Amplification and activating mutations of receptor tyrosine kinases such as *EGFR* are also frequently implicated in the aberrant activation of the RAS/RAF signaling cascade in CRC, with additional evidence that *RAS* mutations result in resistance to anti-EGFR therapy [4]. The prevalence of various somatic mutations and amplifications converging on the activation of the RAS/RAF signaling cascade in CRC underscores the importance of modulating this pathway for anti-tumor effects [5].

As the *RAS* family is the most frequently mutated class of oncogenes in human tumors (33%), considerable effort has focused on the development of RAS inhibitors, though with limited success [6]. The RAF kinases are known downstream effectors of RAS signaling, therefore research has shifted to the identification of inhibitors of RAF kinases and their downstream effectors, leading to the identification of multi-kinase inhibitors [7]. Selective BRAF inhibitors targeting the BRAF^V600E^ mutant have been extensively studied and are effective in melanoma harboring this mutation [8]. The remarkable results in metastatic melanoma spurred interest in the testing of these inhibitors in CRC models also harboring the *BRAF*^V600E^mutation, albeit with limited success and with resistance inevitably occurring in both preclinical and clinical settings [9, 10]. Selective BRAF inhibitors have been shown to exhibit limited anti-proliferative activity in preclinical models of *BRAF*^mut^ CRC as monotherapy [11]. Additionally, a phase II pilot study of vemurafenib in CRC patients with *BRAF*^mut^ disease also concluded that the selective BRAF inhibitor had limited efficacy in this subset of CRC patients [12]. Lack of efficacy of BRAF-selective inhibitors in CRC is largely attributed to resistance mediated by feedback activation of EGFR [13]. Furthermore, in *RAS*^mut^/*BRAF*^WT^ cell lines, BRAF inhibitors do not show efficacy [8] and, rather, this class of drugs induces so-called paradoxical activation of the MAPK pathway in a *RAS*^mut^ background, which could result in enhanced proliferation [14, 15]. The paradoxical MAPK activation in *RAS*^mut^/RAF^WT^ cells has been attributed to several potential mechanisms, including inhibitor-induced relief of the RAF^WT^ autoinhibitory loop [16]. More studies focus on induction of either BRAF^WT^-CRAF^WT^ heterodimerization in non-*BRAF*^mut^ backgrounds [14] and CRAF^WT^ homodimerization [17], which subsequently activate the MEK/MAPK signaling cascade. Another model of paradoxical activation of the MAPK pathway is attributed to RAS-independent transactivation of RAF homodimers or heterodimers due to drug binding and to inhibiting only one dimer partner while the other is transactivated [17, 18]; a panRAF inhibitor could potentially overcome both models of paradoxical activation [18, 19].

A potential mechanistic approach of preventing the paradoxical activation of MAPK in a *RAS*^mut^/*RAF*^WT^ background is to simultaneously inhibit both BRAF and CRAF, as well as the RAF dimer [20]. We have previously identified LY3009120, a type IIa kinase inhibitor that potently inhibits ARAF, BRAF, CRAF kinases and RAF dimers [18]. LY3009120 demonstrated minimal paradoxical MAPK activation in *NRAS*^mut^ and *KRAS*^mut^ settings and was active against *BRAF*^V600E^ and *RAS*^mut^ melanoma, lung and colon cancer cell lines and *in vivo* models [15, 18]. As an extension of this previously published work [14], this report focused on expanded studies of the effects of the panRAF inhibitor LY3009120 on a multitude of preclinical models of human CRC harboring activating mutations in the *KRAS* or *BRAF* genes, including investigation of the effects of LY3009120 on downstream effectors of the RAS/RAF pathway additional to MEK/ERK/RSK. LY3009120 reduced RAF/MEK/ERK signaling and inhibited proliferation of *BRAF*^mut^ and *KRAS*^mut^ CRC lines by inducing G1 cell cycle arrest. In addition to pharmacological inhibition, molecular inhibition of the RAF isoforms by simultaneous siRNA-mediated knockdown of *A-*, *B-* and *CRAF* confirmed the involvement of all three RAF isoforms in the proliferation of *KRAS*^mut^ CRC. Furthermore, LY3009120 suppressed CRC tumor growth in *in vivo* models of CRC. We also investigated potential resistance mechanisms to LY3009120 in a *KRAS*^mut^ background and our findings suggest potential combination opportunities with LY3009120.

## RESULTS

### Effects of LY3009120 on proliferation of CRC cell lines

**Figure 1:**
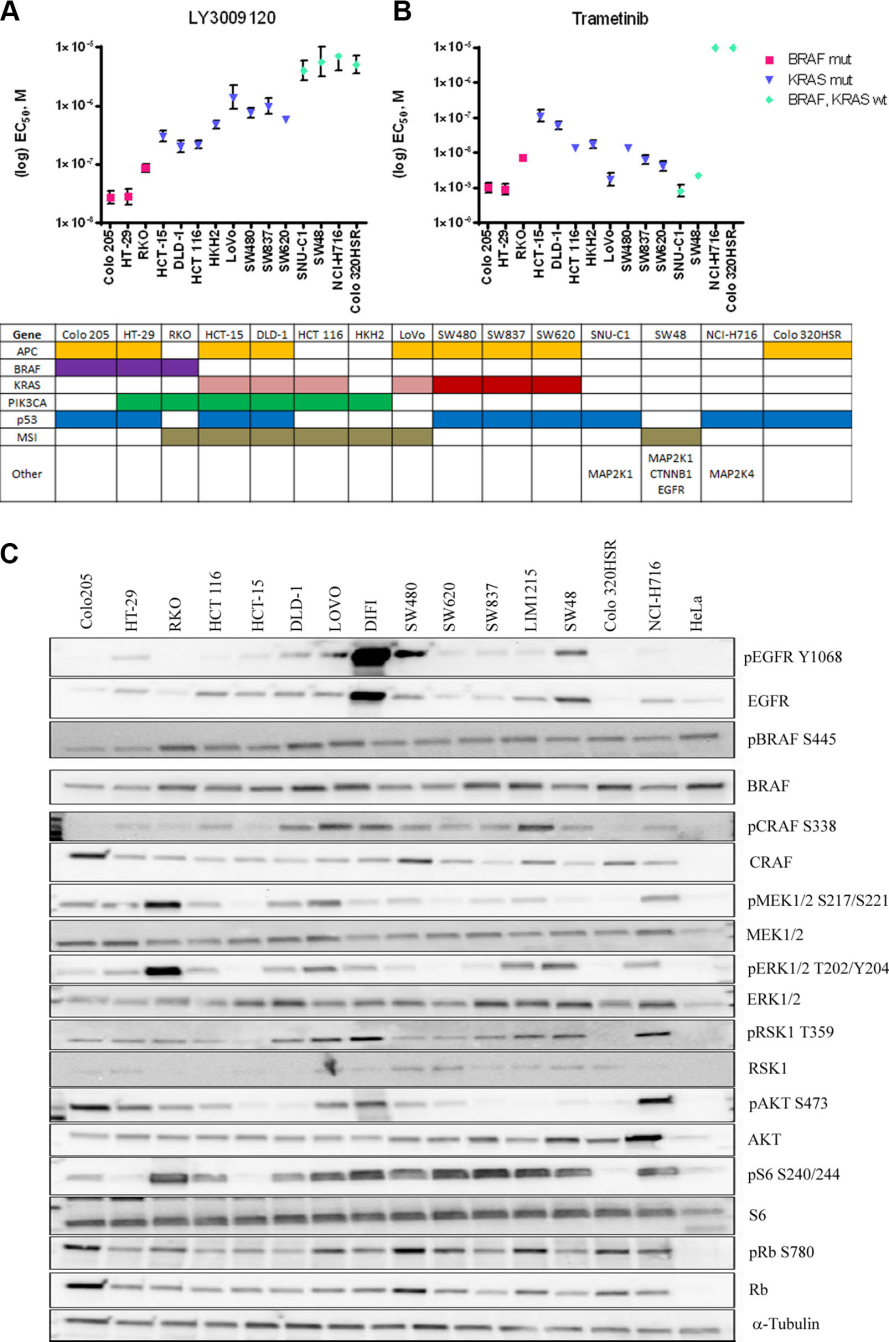
Effects of LY3009120 and trametinib on colorectal cancer cell lines CRC lines were treated with increasing concentrations of LY3009120 (**A**) or trametinib (**B**) and proliferation was assessed at 72 hrs post-treatment by CTG analysis. EC_50_ values were calculated from at least 2 independent experiments with duplicate technical replicates (1 experiment for SW48-trametinib) and are plotted based on the sensitivity and mutational status of each assayed cell line as obtained from COSMIC, as depicted in the table below each graph. Colors denote presence of gene mutation or microsatellite (MSI) instability and *KRAS*^G13^ and *KRAS*^G12^ are depicted by pink and red colors respectively. Other mutations include mutations in the genes *MAP2K1* (SNU-C1 and SW48), *CTNNB1, EGFR* (SW48) and *MAP2K4* (NCI-H716). (**C**) Whole cell lysates of various CRC cell lines were analyzed by Western blot for baseline pathway activation using antibodies against the proteins indicated. The cell lines are oriented in decreasing sensitivity to LY3009120.

We assessed the effects of LY3009120 on a panel of CRC cell lines based on *BRAF*^mut^ and *KRAS*^mut^ background. Biochemical assay data demonstrated inhibition of BRAF^V600E^, BRAF^WT^ and CRAF^WT^ with IC_50_ values of 5.8, 9.1 and 15 nM respectively [15]. In the whole-cell based KiNativ assay measuring the affinity of LY3009120 to each RAF isoform, the IC_50_ was similar among the three RAF isoforms, specifically, 44, 31-47 and 42 nM for ARAF, BRAF and CRAF respectively [15]. Based on the biochemical and KiNativ assay results, we examined the anti-proliferative effects of LY3009120 using concentrations of low nM up to 10 μM. The anti-proliferative effects of LY3009120 assayed by CellTiter Glo (CTG) and EC_50_ values were plotted according to the mutational status of each cell line. Cell lines harboring *BRAF*^V600E^ mutations were the most sensitive to LY3009120, followed by cell lines harboring *KRAS*^G13^ and *KRAS*^G12^ mutations (Figure 1A). There was a >5-fold difference between the most sensitive lines (*BRAF*^mut^ and *KRAS*^mut^) and the least sensitive lines (*KRAS*^WT^/*BRAF*^WT^) (Figure 1A). Of note, sensitivity of CRC cell lines to LY3009120 could not be predicted based on the activation status of the MAPK and PI3K signaling cascades, but rather appeared to be a function of the *BRAF* and *KRAS* mutational status (Figure 1A and 1C). For example, the cell line SW480 (*KRAS*^mut^) was slightly less responsive to LY3009120 than LoVo (*KRAS*^mut^) despite SW480 exhibiting lower levels of pERK1/2 (Figure 1C).

Similarly, treatment with the MEK1/2 inhibitor trametinib indicated a modest difference in sensitivity between the *BRAF*^mut^ and *KRAS*^mut^ cell lines, with *KRAS*^mut^ cell lines being slightly less sensitive than the *BRAF*^mut^ cell lines (Figure 1B). Interestingly two *KRAS*^WT^/*BRAF*^WT^ cell lines, SNU-C1 and SW48, which were among the least sensitive to LY3009120 treatment exhibited increased sensitivity to trametinib (Figure 1B). SW48 harbors an *EGFR*^G2155A^ mutation and various *MAP2K1* mutations [21] while SNU-C1 has an activating *MAP2K1*^F53L^ mutation [22, 23].

### Effects of LY3009120 on cell signaling and gene expression

**Figure 2:**
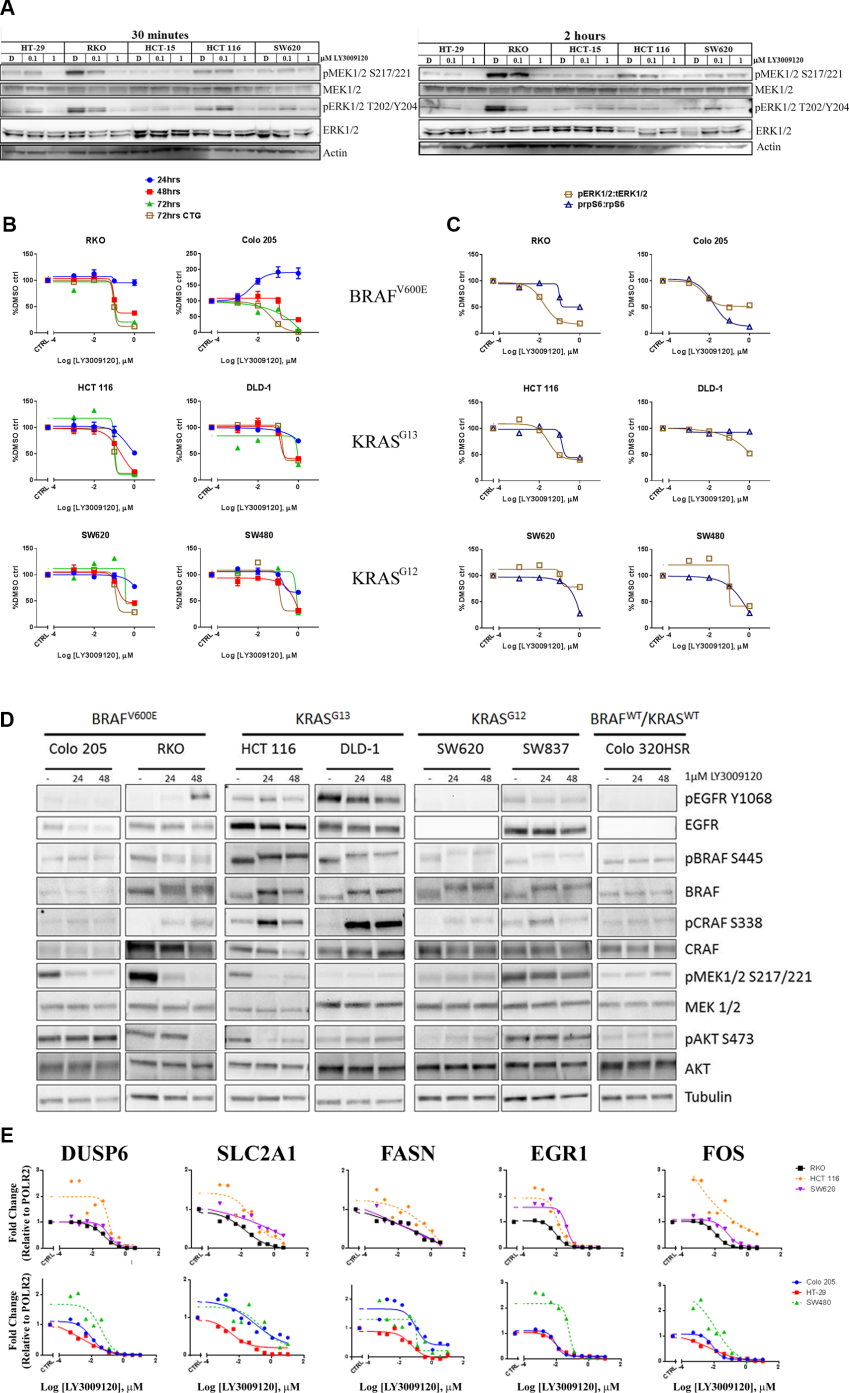
Effects of LY3009120 on signaling and gene expression (**A**) CRC cell lines were treated with DMSO, 0.1 μM or 1 μM LY3009120 for 30 minutes (*left panel*) or 2 hrs (*right panel*). Whole cell lysates were analyzed by Western blot using antibodies against the proteins indicated. (**B**) CRC cell lines were treated with increasing concentrations of LY3009120 and fixed at the times indicated. Cells were stained with Hoechst stain and nuclei counts were obtained. At the 72 hr time point, replicate plates were assessed for proliferation by CTG analysis. (**C**) CRC cell lines were treated with DMSO or logarithmic concentrations of LY3009120 and fixed at 24 hrs post-treatment. Cells were stained for immunofluorescence analysis with Hoechst stain and antibodies against the proteins indicated and the average intensity of the signal for each analyte was measured by HCI. Results are plotted as percent of DMSO-treated cells and are representative of two independent experiments. The signal for pERK1/2 T202/Y204 and pS6 S240/244 was normalized to the levels of ERK1/2 and S6 respectively. (**D**) CRC cell lines were treated with DMSO or 1 μM LY3009120 for 24 and 48 hrs. Whole cell lysates were analyzed by Western blot using antibodies against the proteins indicated. (**E**) CRC cell lines were treated with DMSO or increasing concentrations of LY3009120 for 24 hrs. Expression levels of the indicated genes were assessed by Affymetrix analysis.

In order to confirm inhibition of the MAPK pathway, we examined the phosphorylation of MEK1/2 and ERK1/2 following a 30 minute (*left panel*) or 2 hour (*right panel*) treatment with LY3009120 (Figure 2A, *left* and *right panels*, respectively). Potent inhibition of phosphorylation of both MEK1/2 and ERK1/2 was observed with 1 μM LY3009120 treatment in cell lines with high basal levels of pMEK1/2 and pERK1/2 (RKO and HCT 116) at both time points. No major deviations from baseline were observed in HCT-15 and SW620, both of which exhibited markedly lower baseline phosphorylation than the aforementioned cell lines (Figure 2A). HT-29, which has moderate basal pMEK1/2 and pERK1/2 signaling also showed decreased phosphorylation upon treatment (Figure 2A) Interestingly, in both HCT 116 and SW620, a minor increase in pERK1/2 is observed upon 0.1 μM treatment; however, this minor increase was abolished upon treatment with higher concentrations of the inhibitor (Figure 2A).

We further characterized the effects of LY3009120 on CRC cell lines of *BRAF* (V600) and *KRAS* mutational status (G13 and G12) using high content imaging (HCI), as previously described [24]. Assessment of nuclei counts demonstrated that LY3009120 reduced proliferation of cell lines harboring *BRAF* and *KRAS* mutations in a time- and concentration-dependent manner (Figure 2B). Inhibition of proliferation was most evident at 72 hrs, at which time we also evaluated the anti-proliferative effects of LY3009120 by CellTiter Glo (CTG). The anti-proliferative effects of LY3009120 were consistent between the two methods for all cell lines assessed (Figure 2B). A slight increase in proliferation of Colo 205 was observed at 24 hrs, however, this result was not consistent across the other time points.

Replicate plates were assessed for the effects of LY3009120 on the MAPK pathway at 24 hrs post-treatment, using the ratio of pERK1/2 T202/Y204:total ERK1/2 as an output. A reduction in pERK1/2:total ERK1/2 was observed in the majority of the cell lines assayed (Figure 2C). Although not an ERK1/2 mediated phosphorylation event, a decrease in the phosphorylation of ribosomal protein S6 (“S6”) at residues S240/244 is implicated in the responsiveness to selective BRAF inhibition in *BRAF*^mut^ melanoma cells [25], therefore we examined the effects of LY3009120 on this phosphorylation event in CRC. LY3009120 treatment reduced the pS6 S240/244:total S6 ratio in both *BRAF*^mut^ and the majority of *KRAS*^mut^ cell lines tested (Figure 2C). In this system, however, the decrease of pS6 S240/244:total S6 ratio did not appear to be predictive of growth sensitivity to LY3009120. As observed from the comparison of HCT 116 with RKO and SW620, while HCT 116 cells were more sensitive to LY3009120 treatment than SW620, the inhibition of pS6 S240/244:total S6 in SW620 cells was more pronounced; similarly, the inhibition of pS6 S240/244:total S6 was comparable between RKO and HCT 116, despite the latter being less sensitive to LY3009120 treatment (Figure 2B). Additional comparison of DLD-1 with SW480 also indicated that inhibition of pS6 S240/244:total S6 is not predictive of sensitivity to LY3009120 (Figure 2C). We further characterized the effects of LY3009120 on other effectors of the RAS/RAF and PI3K pathways by Western blot, as antibodies suitable for high content imaging were not available. Concomitant with inhibition of pERK1/2, observed by HCI, panRAF inhibition abolished the phosphorylation of MEK1/2 in Colo 205, RKO and HCT 116, while the phosphorylation levels of MEK1/2 were unchanged in DLD-1, SW620 and SW837 (Figure 2D). The levels of pBRAF S445 were largely unchanged across all of the cell lines; however, panRAF inhibitor treatment induced a mobility shift of BRAF, particularly evident in the *KRAS*^mut^ cell lines (Figure 2D). Additionally, levels of the activating phosphorylation of CRAF (pCRAF S338) increased with treatment in the majority of *BRAF*^mut^ and *KRAS*^mut^ cell lines; however, this did not result in increased pMEK1/2 levels, providing evidence that the kinase activity of CRAF is impaired in the presence of the inhibitor (Figure 2D). As increased EGFR signaling is associated with and found to mediate resistance to BRAF inhibitor therapy in CRC [13, 26], we examined the effect of LY3009120 on EGFR activation and total EGFR levels. For the majority of cell lines, changes in the phosphorylation of EGFR were unremarkable, with the exception of the RKO cell line where pEGFR Y1068 was increased at the 48 hr timepoint and in HCT 116 where a modest increase was observed at the 24hr timepoint but was diminished with prolonged treatment (Figure 2D).

As the MAPK pathway is known to regulate gene expression [27, 28], we also assessed the effects of LY3009120 on the expression of a subset of genes associated with pathway activation, such as *DUSP6*, *EGR1*, *FOS*, *SLC2A1* and *FASN*. Treatment with LY3009120 resulted in a concentration-dependent decrease in expression of all the genes assayed (Figure 2E). Interestingly, at LY3009120 concentrations below 10 nM, there was a slight induction of *DUSP6*, *EGR1* and *FOS* in some cell lines, notably the *KRAS*^mut^ cell lines SW480 and HCT 116, and the effect was mitigated by concentrations closer to the EC_50_ for anti-proliferation (Figure 2E).

### Effects of LY3009120 on cell cycle and apoptosis

**Figure 3:**
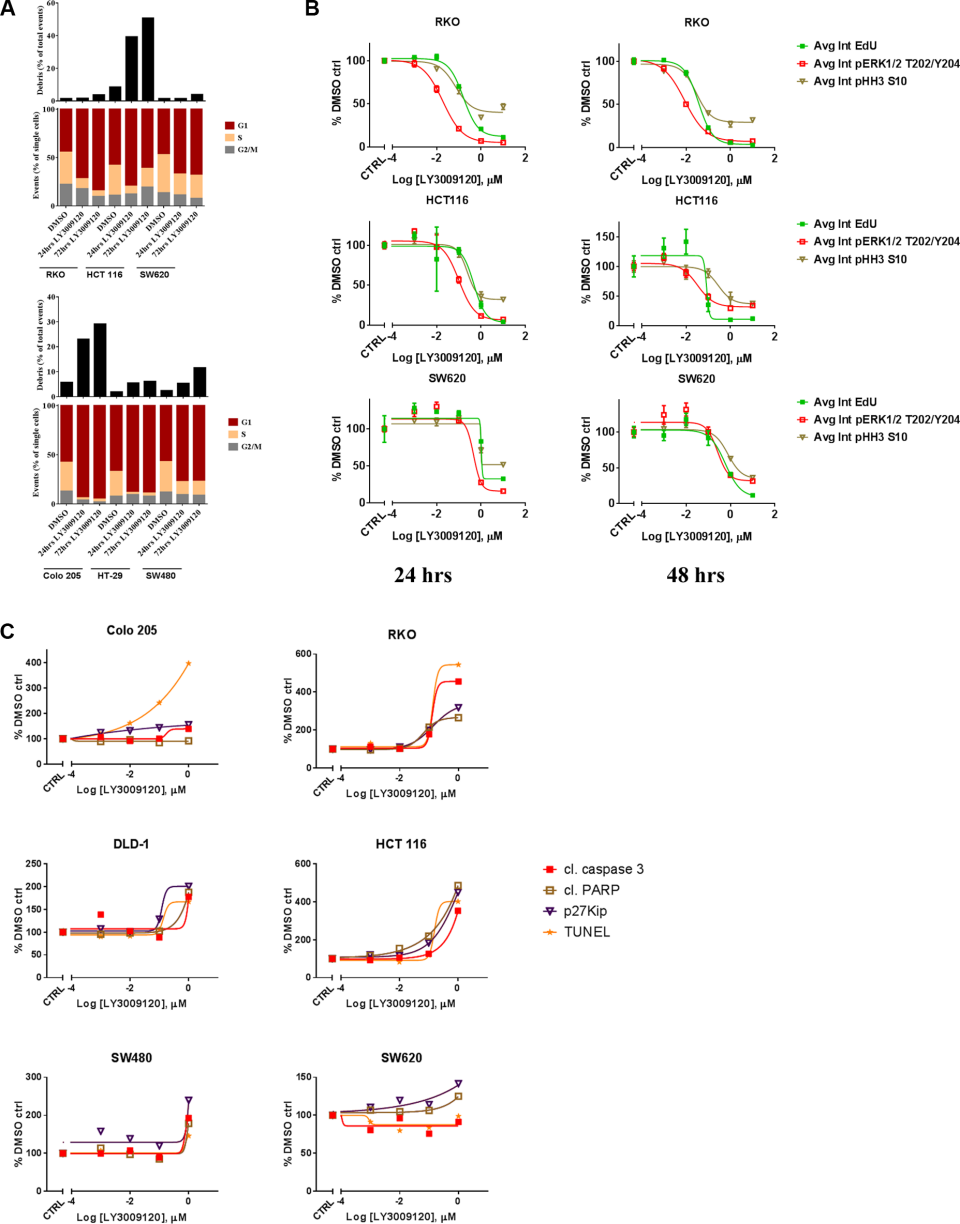
Effects of LY3009120 on cell cycle and apoptosis (**A**) Representative experiment of CRC cell lines treated with either DMSO or LY3009120 (0.5 μM) for the times indicated, fixed and stained with propidium iodide and analyzed for cell cycle by flow cytometry. (**B**) CRC cell lines were treated with increasing concentrations of LY3009120, fixed at 24 or 48 hrs (*left* and *right* panels respectively) and stained for immunofluorescence with Click-iT^®^ EdU and antibodies against pERK1/2 T202/Y204 and pHH3 S10 as indicated. The average intensity of the signal for each analyte was measured by HCI. The data are representative of two independent experiments each conducted in triplicate technical replicates, with results plotted as percent of DMSO-treated cells. (**C**) Cells were treated with increasing concentrations of LY3009120 and fixed at 48 hrs post-treatment. Cells were stained for immunofluorescence analysis with antibodies against the proteins indicated and the average intensity of the signal for each analyte was measured by HCI. Results are plotted as percent of DMSO-treated cells and are representative of two independent experiments.

Increased progression through the cell cycle is one of the hallmarks of cancer [29] and as the RAS/RAF/MAPK pathway is implicated in cell cycle progression [30] we examined whether the anti-proliferative effects of LY3009120 could be attributed to cell cycle arrest. Treatment of both *BRAF*^mut^ and *KRAS*^mut^ CRC cell lines with LY3009120 induced an increase in the percentage of cells in G1, indicative of G1 cell cycle arrest, with significant debris accumulation (sub-G1 population) in the HCT 116 and Colo 205 cell lines, indicating potential induction of apoptosis (Figure 3A). We further confirmed the LY3009120-mediated perturbation of cell cycle by simultaneous assessment of EdU, pHH3 S10 and pERK1/2 T202/Y204 in a *BRAF*^mut^ and two *KRAS*^mut^ CRC lines (Figure 3B and Supplementary Figure S1). Treatment with LY3009120 resulted in a concentration dependent decrease in average EdU and pHH3 S10 intensity as early as 24 hrs post-treatment, indicating a decrease in S and G2/M cell cycle phases respectively (Figure 3B and Supplementary Figure S1), complementing our G1 cell cycle arrest observations [31]. Additionally, the decrease in EdU and pHH3 S10 is proportional to the decrease in pERK1/2 staining and LY3009120-sensitivity (Figure 3B and Supplementary Figure S1). The activity of p27, a known regulator of G1-progession, is negatively regulated by the MAPK pathway [32] and therefore we examined the effects of LY3009120 on p27 levels. Treatment with LY3009120 resulted in an increase in p27 expression in all cell lines, albeit at variable levels of induction (Figure 3C and Supplementary Figure S2).

Accumulation of debris identified by flow cytometry could signify apoptotic cells [33], prompting us to investigate the effects of LY3009120 on various apoptotic markers. A concentration-dependent increase in TUNEL (late apoptosis) and cleaved caspase-3 staining (early apoptosis) were more prominent in the *BRAF*^mut^ and *KRAS*^G13^ cell lines, while *KRAS*^G12^ cell lines presented with only a modest induction of these analytes at the highest concentration of LY3009120 tested (Figure 3C and Supplementary Figure S2). Detection of cleaved PARP was more evident in HCT 116 cells, while the remaining cell lines presented with variable levels of induction of PARP cleavage (Figure 3C and Supplementary Figure S2).

### Simultaneous knockdown of A-, B- and CRAF inhibited proliferation of KRAS-mutant cell lines

**Figure 4:**
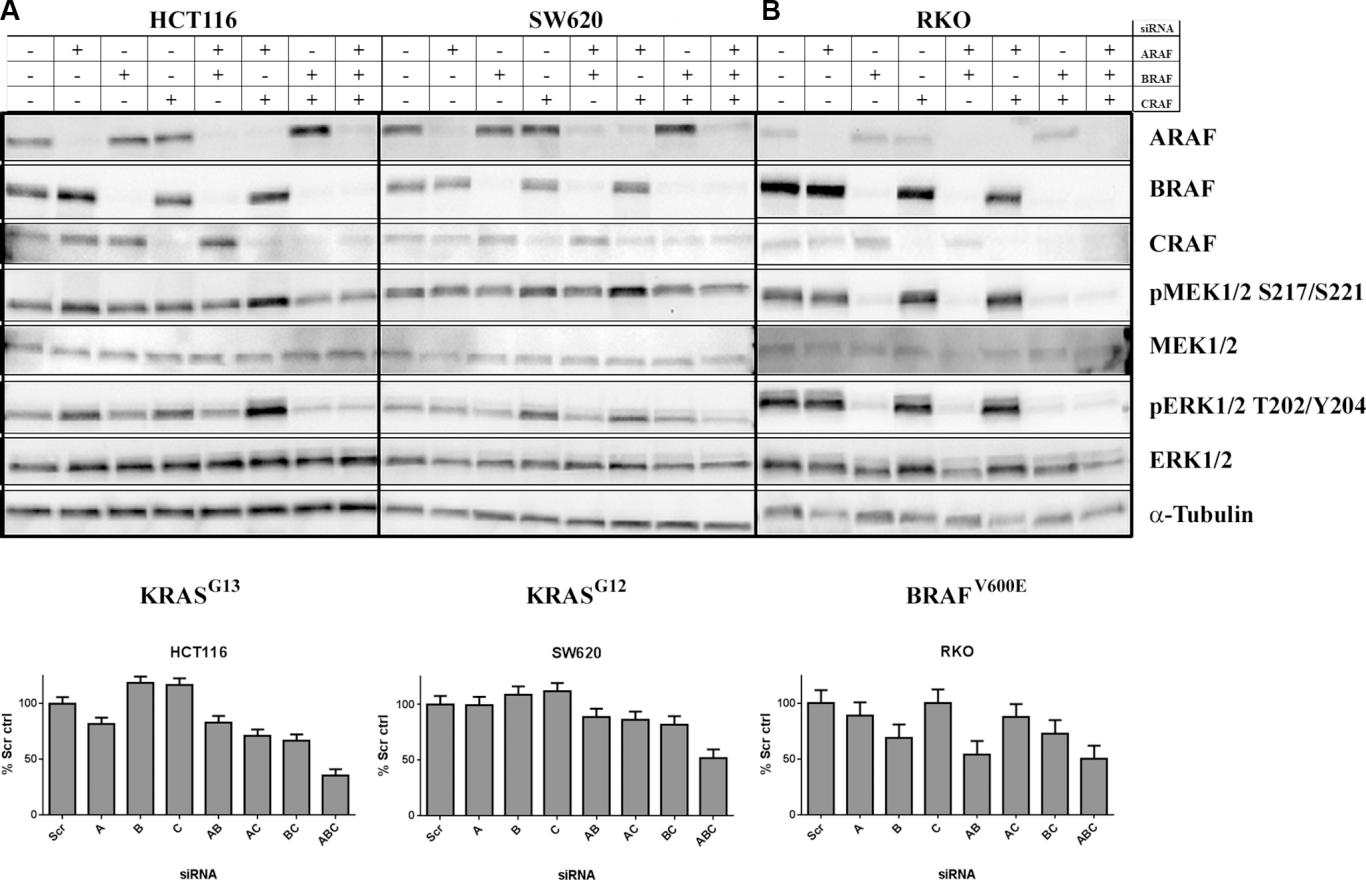
Concomitant knockdown of *A, B* and *CRAF* induces anti-proliferative effects in *KRAS*^mut^ CRC lines *Upper panels:* (**A**) *KRAS*^mut^ (HCT 116 and SW620) or B, *BRAF*^mut^ (RKO) CRC cell lines were transfected with the indicated siRNAs. Whole cell lysates were isolated at 72 hrs post-transfection and were analyzed by Western blot using antibodies against the proteins indicated. *Lower panels:* A, *KRAS*^mut^ (HCT 116 and SW620) or (**B**) *BRAF*^mut^ (RKO) CRC cells were transfected with the indicated siRNAs. Effects of transfection on proliferation were assessed by CTG at 96 hrs post-transfection normalized to non-targeting control. Results represent aggregated data of 2 independent experiments run in six technical replicates each.

As LY3009120 is a panRAF inhibitor, we examined the contribution of each RAF isoform to MAPK pathway activation and proliferation of *KRAS*^mut^ CRC cells. Simultaneous knockdown of all three *RAF* isoforms was obtained in all cell lines examined (Figure 4A and 4B, top panels). The triple knock-down was more potent in inducing anti-proliferative effects than either single or double-isoform knockdown in both a *KRAS*^G13^ (HCT 116, *p* < 0.001) (Figure 4A, *lower left panel* and Supplementary Table S2) and a *KRAS*^G12^ CRC cell line (SW620, *p* < 0.001) (Figure 4A, *lower right panel* and Supplementary Table S3). Simultaneous knockdown of all three isoforms also resulted in a slight reduction of pMEK1/2 and pERK1/2 compared to single knockdown, while simultaneous knockdown of *B* and *CRAF* isoforms decreased pMEK1/2 and pERK1/2 to levels similar to that of the triple knockdown (Figure 4A, *top panels*). In contrast to the observations in the *KRAS* cell lines, in the *BRAF*^V600E^mutant cell line RKO, pMEK1/2 and pERK1/2 as well as proliferation were consistently reduced upon *BRAF* knockdown, either as a single knockdown or in combination with *A* and/or *CRAF* knockdown (Figure 4B and Supplementary Table S4).

### *In vivo* efficacy of LY3009120

**Figure 5:**
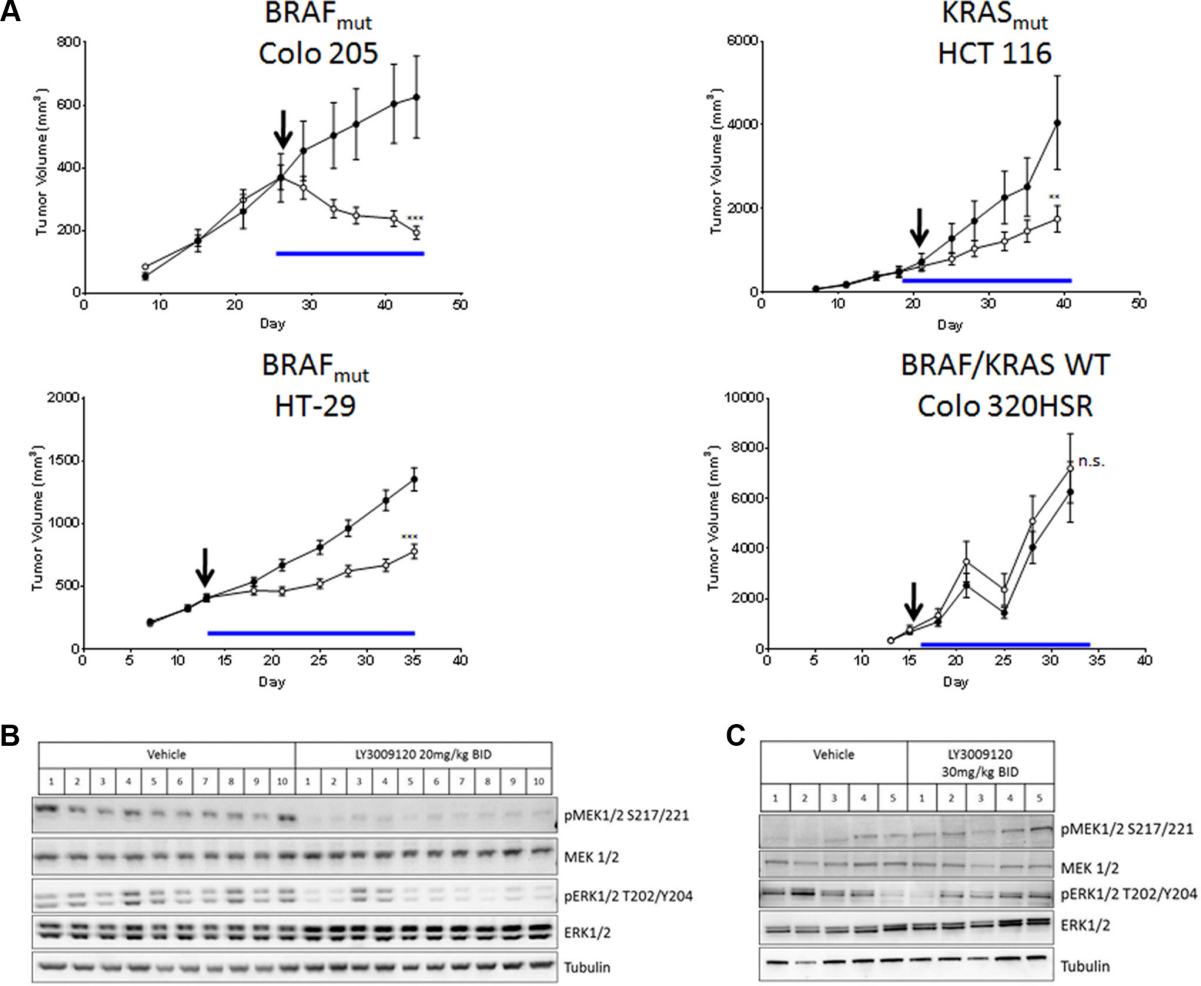
Effects of LY3009120 on CRC xenografts (**A**) Colo 205 (*BRAF*^mut^), HT-29 (*BRAF*^mut^), HCT 116 (*KRAS*^mut^) or Colo 320HSR (*KRAS*^WT^*/BRAF*^WT^) CRC lines were implanted subcutaneously in the right flank of nude rats and the animals were treated orally with either vehicle (•) or LY3009120 (Colo 205: 20 mg/kg; HCT 116: 30 mg/kg; HT-29: 20 mg/kg; Colo 320HSR 30mg/kg) (○) twice daily and were monitored for tumor size. Arrow: Day of dosing initiation. Blue line: Duration of dosing. *p* < 0.01 (**); *p* < 0.001 (***); n.s. (not significant). (**B**) HT-29 (*BRAF*^mut^) xenograft model treated with either vehicle or LY3009120 (20 mg/kg BID) or (**C**) Colo 320HSR xenograft model treated with either vehicle or LY3009120 (30 mg/kg BID) were assessed by Western blot for levels of phospho- and total MEK1/2, ERK1/2 and tubulin as indicated.

Based on the anti-proliferative effects of LY3009120 observed *in vitro*, we examined the *in vivo* efficacy of LY3009120 in CRC xenograft models of various mutational backgrounds. Treatment of Colo 205 xenografts (*BRAF*^mut^) with LY3009120 resulted in statistically significant tumor regression (46.7% regression from baseline, *p* < 0.001) (Figure 5A, *top left panel*), while treatment of HCT 116 xenografts (*KRAS*^mut^) resulted in statistically significant inhibition of tumor growth (delta(T/C)= 35.4%, *p* < 0.01) (Figure 5A, *top right panel*). Examination of an additional *BRAF*^mut^ xenograft model, HT-29, confirmed the *in vivo* efficacy of LY3009120 (delta(T/C)= 40%, *p* < 0.001) in a *BRAF*^mut^ CRC setting (Figure 5A, *lower left panel*), while LY3009120-treatment of Colo 320HSR (*KRAS*^WT^*/BRAF*^WT^) indicated lack of *in vivo* efficacy in a *KRAS*^WT^*/BRAF*^WT^ xenograft model (Figure 5A, *lower right panel*).

Examination of signaling in xenograft models indicated that LY3009120 treatment reduced pMEK1/2 in all HT-29 xenografts and reduced pERK1/2 in the majority of HT-29 xenografts (Figure 5B) while LY3009120 had unremarkable effects on the phosphorylation of MEK1/2 and ERK1/2 in the Colo 320HSR xenograft model (Figure 5C) at a 50% increased dose.

### Resistance of a *KRAS^mut^* cell line to LY3009120

**Figure 6:**
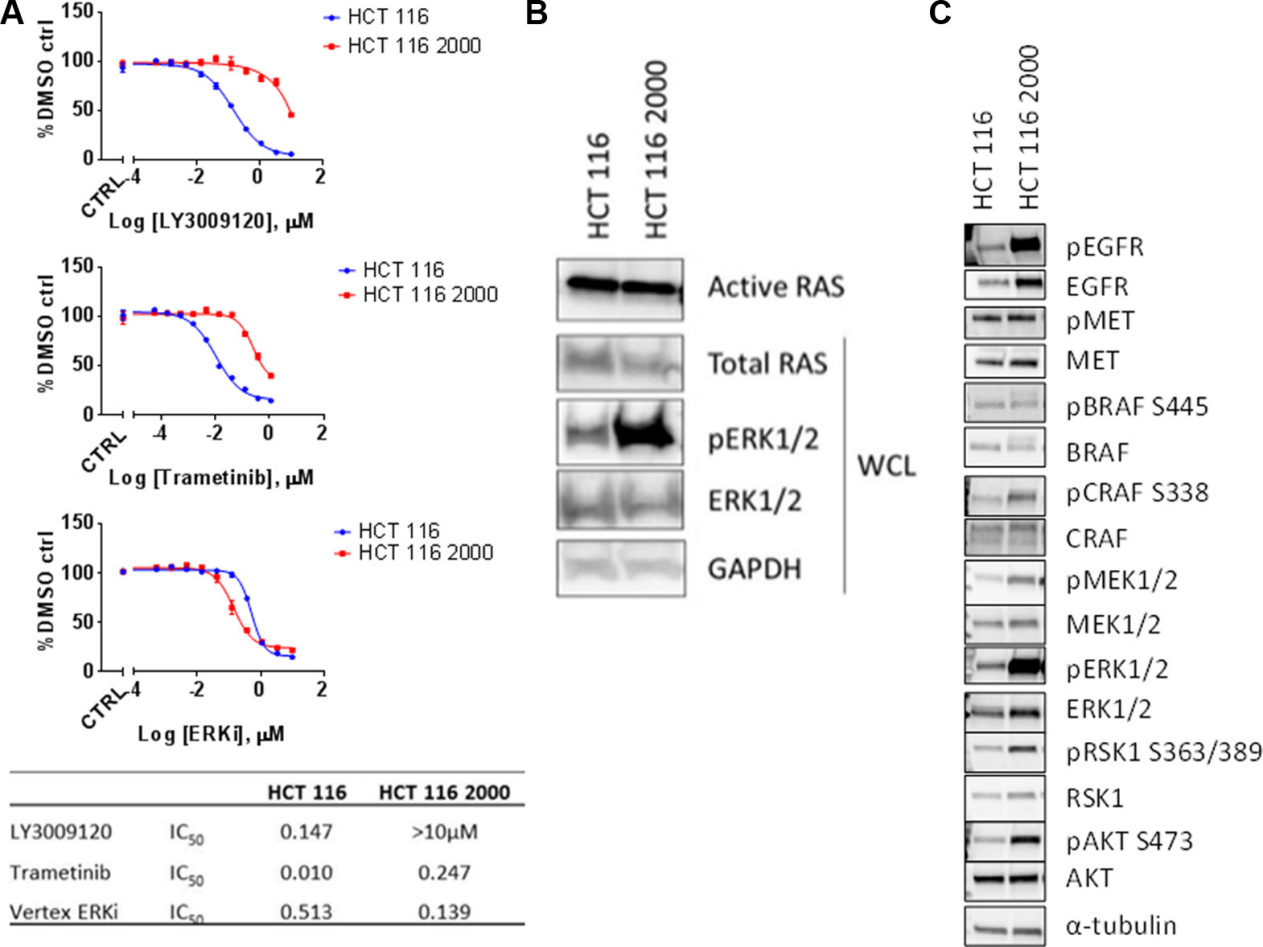
ERK-mediated mechanism of resistance to LY3009120 (**A**) HCT 116 parental (blue line) and HCT 116 LY3009120-resistant cells (red line) were generated and assessed for their sensitivity to various kinase inhibitors of the MAPK signaling cascade. LY3009120: panRAF inhibitor (*top panel*); trametinib: MEK1/2 inhibitor (*middle panel*); ERKi: VX-11e ERK2 inhibitor (*lower panel)*. (**B**) RAS-GTP levels were analyzed from cell lysates of LY3009120-sensitive and LY3009120-resistant HCT 116 by an active-RAS pulldown assay as delineated in the experimental procedures. Whole cell lysates were also analyzed by Western blot using antibodies against the proteins indicated. (**C**) Whole cell lysates of LY3009120-sensitive and LY3009120-resistant HCT 116 were analyzed by Western blot for baseline pathway activation using antibodies against the proteins indicated.

As other selective BRAF inhibitors have been studied in the context of kinase inhibitor resistance [10, 34, 35], we explored potential mechanisms of resistance to LY3009120. We generated a LY3009120-resistant HCT 116 cell line (labeled HCT 116 2000) by continually culturing cells in the presence of LY3009120 (see “Materials and Methods”). Evaluation of *in vitro* resistance by LY3009120 treatment indicated that the resistant cells were at least 100-fold less sensitive to drug (Figure 6A). As resistance to selective BRAF inhibitors can occur via reactivation of the MEK/ERK pathway [36], we assessed the effects of a MEK1/2 inhibitor (trametinib) or an ERK2 inhibitor (VX-11e) on the proliferation of LY3009120-resistant lines. Interestingly, while HCT 116 2000 was largely insensitive to MEK inhibition (Figure 6A, *middle panel)*, with a > 24-fold decrease in sensitivity, this cell line maintained sensitivity to the ERK2 inhibitor, similar to its parental counterpart (Figure 6A, *lower panel*). Examination of response to other internal and external ERK inhibitors yielded similar trends (data not shown).

As RAS hyperactivation is also known to contribute to BRAF inhibitor resistance [34], we evaluated RAS activity in the LY3009120-resistant cell line. Active RAS pull-down experiments indicated that RAS activity in the HCT 116 2000 line was similar to parental HCT 116 RAS activation levels; however, a robust increase of pERK1/2 was observed (Figure 6B). Based on the changes in the phosphorylation status of ERK1/2, we further evaluated the levels of various signaling effectors that could be involved in resistance to LY3009120. Examination of pEGFR indicated an increase in phosphorylation of this receptor in HCT 116 2000 cells compared to its parental counterpart, despite changes in the EGFR receptor levels, while changes in the phosphorylation of MET were unremarkable (Figure 6C). As in Figure 6B, HCT 116 2000 cells presented with RAF/MEK/ERK pathway hyperactivation relative to their parental counterpart, as indicated by the phosphorylation status of BRAF, CRAF, MEK1/2, ERK1/2 and RSK1; similar differences were observed in the phosphorylation status of AKT, indicating an increase in AKT signaling (Figure 6C).

## DISCUSSION

The clinical efficacy of selective BRAF inhibitors against *BRAF*^V600E^ mutant melanoma patients inspired interest in CRC since somatic mutation of this gene occurs in ∼5–10% of colon cancer patients and is associated with poor prognosis [3]. However, feedback activation of EGFR upon BRAF blockade in *BRAF*^mut^ CRC cells [13] results in a minimal response to selective BRAF inhibitors with only 5% of patients responding, compared to an 80% response rate in *BRAF*^V600E^ melanoma patients [37]. This low response rate is due in part to differential expression/activation of EGFR in colorectal cancer compared to melanoma, ultimately leading to MAPK pathway activation and thereby circumventing BRAF inhibition[13]. In addition to the underwhelming response of *BRAF*^V600E^ CRC to selective BRAF inhibition, such inhibitors are contraindicated in *KRAS*^WT^*/BRAF*^WT^ and *KRAS*^mut^/*BRAF*^WT^ backgrounds due to the RAS-mediated paradoxical activation of MAPK [38]. The prevalence of *KRAS* mutations in CRC, which are mutually exclusive from *BRAF* mutations [39], also underscores the need to identify diverse means of targeting the RAS/RAF/MEK/ERK signaling cascade [38].

Because of the prevalence of *KRAS* and *BRAF* mutations in CRC [39], as well as the involvement of the aforementioned mutations in acquired resistance to therapy [40] we hypothesized that a panRAF inhibitor like LY3009120 would have preclinical activity in this tumor setting. Previously, LY3009120 was extensively studied in *RAS*^mut^ and *BRAF*^mut^ melanoma preclinical models with evidence of activity in *RAS*^mut^ lung cancer and CRC models [15]. In this study, we expanded on the effects of LY3009120 in preclinical CRC models so as to examine the efficacy of this molecule in this tumor type. LY3009120 exhibited anti-proliferative effects in both *KRAS*^mut^ and *BRAF*^mut^ CRC cell lines, but had limited efficacy in *KRAS*^WT^*/BRAF*^WT^ cell lines. Furthermore, within the *KRAS*^mut^ CRC setting, we did not observe significant differences in the anti-proliferative effects of LY3009120 between the G13 and G12 mutants, though a *trend* towards increased sensitivity of G13 mutants was observed. Consistent with previous studies with MEK inhibitors in CRC, sensitivity to LY3009120 was dictated by *KRAS*/*BRAF* mutational status rather than baseline ERK1/2 activation [21]. Additionally, one of the *KRAS*^WT^*/BRAF*^WT^ cell lines that was sensitive to MEK inhibition but insensitive to LY3009120 harbors an activating MEK mutation, suggesting that the anti-proliferative mechanism of action of LY3009120 in CRC is mediated in part by the MEK/ERK signaling cascade.

Sensitivity of *BRAF*^mut^ melanoma cells to RAF and MEK inhibition has been associated with decreased pS6 S240/244 phosphorylation [25] and we similarly observed a LY3009120-concentration dependent decrease in pS6 in *KRAS*^mut^ and *BRAF*^mut^ CRC lines. This observation, however, is not likely associated with LY3009120 sensitivity as the relatively LY3009120-insensitive SW480 and SW620 cell lines presented with a greater decrease in pS6 than the more sensitive HCT 116 and DLD-1 lines. The lack of association of this mTORC1-dependent phosphorylation event with response to LY3009120 further supports the differentiation of the effects of RAF inhibitors on preclinical CRC models as compared to preclinical melanoma models, where sensitivity to BRAF- and MEK-specific inhibitors was positively correlated with a decrease in pS6 phosphorylation [25, 41].

In addition, LY3009120 treatment caused a mobility shift in BRAF, which was more evident in the *KRAS*^mut^ cell lines. Interestingly, a mobility shift was previously reported in an *NRAS*^mut^ melanoma cell line treated with a BRAF inhibitor and was attributed to a phosphorylation event that was both MEK pathway-dependent and independent [19]. As RAF inhibitors, including LY3009120, have been reported to induce RAF dimerization in *RAS*^mut^ settings [15, 17, 42], the phosphorylation event associated with a mobility shift of BRAF could be implicated in dimer dynamics. Moreover, a rebound activation of MEK/ERK was not persistent upon treatment with LY3009120, thereby differentiating this compound from BRAF selective inhibitors that have been shown to be ineffective in *BRAF*^mut^ CRC due to EGFR-mediated activation of MEK/ERK through activation of the RAS-CRAF axis [26].

Consistent with the involvement of the RAS/RAF/MEK/ERK signaling cascade in cell cycle progression [30] and previous studies of LY3009120 in melanoma [15], the anti-proliferative effects of LY3009120 in CRC were associated with G1 cell cycle arrest and isolated induction of cell death in both *BRAF*^mut^ and *KRAS*^mut^ CRC cell lines. Additionally, our siRNA experiments using simultaneous knockdown of *A*, *B* and *CRAF* confirmed that panRAF inhibition is superior to single or double RAF knockdown in a *RAS*^mut^ background, as knockdown of all three genes was more effective in inducing an anti-proliferative response than either single or double RAF knockdown in the *KRAS*^mut^ cell lines HCT 116 and SW620. The siRNA studies, therefore, confirmed the anti-proliferative response to the panRAF inhibitor LY30091200. Surprisingly, in isolated experiments, knockdown of *BRAF* or *CRAF* resulted in increased pMEK1/2 and pERK1/2 levels compared to control, however, this was not a reproducible observation. In contrast to *KRAS*^mut^ CRC cell lines, proliferation and survival of *BRAF*^mut^ CRC cells are mainly driven by the *BRAF*^mut^ gene [43], therefore, as expected, *BRAF* knockdown affected the proliferation and pERK1/2 levels in the *BRAF*^mut^ CRC cell line RKO.

In our animal studies, LY3009120 was efficacious against both *BRAF*^mut^ and *KRAS*^mut^, but not *KRAS*^WT^*/BRAF*^WT^ CRC xenograft models. Examination of MEK/ERK signaling in resected tumors also indicated that LY3009120 was effective in inhibiting pMEK1/2 and pERK1/2 *in vivo* only in the *BRAF V600E* model.

As resistance to targeted agents such as BRAF and EGFR inhibitors remains a significant clinical problem in CRC [4, 10, 13, 44], we also investigated potential mechanisms of resistance to LY3009120 using a *KRAS*^mut^ CRC cell line. While extensive experiments regarding potential mechanisms of resistance to LY3009120 are beyond the scope of this manuscript, over the course of characterizing the effects of LY3009120 on CRC models we developed a LY3009120-resistant cell line. The cell line maintained resistance to the MEK inhibitor trametinib in addition to the LY3009120 inhibitor but remained sensitive to ERK inhibition, alluding to an ERK-dependent mechanism of resistance. Interestingly, examination of signal transduction pathways indicated a hyperactivation of upstream signaling pathways such as EGFR, MEK and PI3K/AKT in the *KRAS*^mut^ cell line suggesting potential combinational strategies of LY3009120 with inhibitors targeting these effectors. In addition, our findings of reactivation of the MEK/ERK pathway in the LY3009120-resistant cell line are in line with recent studies examining clinical acquired resistance of *BRAF*^mut^ CRC to RAF inhibitor combinations due to genetic alterations in the MAPK pathway [9]. Furthermore, a recent study examining resistance to selective RAF inhibitors [45], concluded that combination of pan-RAF and MEK inhibitors overcame such resistance, thus strengthening the argument for future combinational studies. Moreover, in *BRAF*^mut^ melanoma, combination of an AKT inhibitor with a BRAF-selective inhibitor reportedly reversed the resistance to either inhibitor [46]. Given the increased phosphorylation of AKT in the resistant line compared to its parental counterpart, combination of LY3009120 with an AKT inhibitor could also be explored for overcoming potential resistance to LY3009120. Generation of *BRAF*^mut^ CRC lines that are resistant to LY3009120 will also help determine whether resistance to LY3009120 occurs through the same mechanism or if it is defined by the *BRAF*/*KRAS* mutational status.

The cross talk between RAF/MEK/ERK and PI3K/AKT pathways has been well characterized, as well as their common regulation by RAS [47], with feedback activation of one of the pathways occurring upon inhibition of the other [48]. In *KRAS*^mut^ backgrounds, receptor tyrosine kinases (RTKs) exert dominant control over PI3K signaling, while KRAS appears to mediate MEK/ERK pathway regulation [49]. As a result, combination strategies of LY3009120 with PI3K/AKT targeted inhibitors could also be explored in LY3009120-sensitive CRC models and should be compared to the effects of MEK inhibition in combination with PI3K/AKT inhibitors. Studies of PI3K inhibition in a *KRAS*^mut^*/PIK3CA*^WT^ CRC model demonstrated that due to MAPK activation PI3K inhibition alone was insufficient in inducing anti-proliferative effects, thereby alluding to the significance of the RAF/MAPK pathway in the proliferation of *KRAS*^mut^*/PIK3CA*^WT^ CRC [50]. Combination of PI3K inhibition with MEK inhibition overcame PI3K-inhibitor resistance [50] and it is therefore plausible that similar effects could be observed with LY3009120/PI3K inhibitor combinations. A similar rationale may be employed for comparison of MEK/AKT-inhibitor and LY3009120/AKT-inhibitor combinations in *KRAS*^mut^*/PIK3CA*^WT^ CRC models, with potentially similar outcomes to the PI3K combinations. As *PIK3CA* mutations often coexist with *KRAS* and *BRAF* mutations in advanced cancers, including colorectal cancers [51], combination of AKT inhibition with panRAF inhibition are expected to induce at a minimum additive effects in both *KRAS*^mut^*/PIK3CA*^mut^ and *BRAF*^mut^*/PIK3CA*^mut^ CRC models.

In conclusion, our data established that LY3009120, a selective panRAF inhibitor, is superior to previously investigated selective BRAF inhibition in preclinical models of human CRC, as it potently inhibited proliferation and tumor growth in the *BRAF*^mut^ and *KRAS*^mut^ subtypes. The underwhelming response of CRC to selective BRAF inhibitors along with the paradoxical activation of such selective BRAF inhibitors in a *RAS*^mut^ setting underscores the need for novel approaches to abrogate signaling through the RAF/MEK/ERK pathway in *KRAS*^mut^ CRC.

## MATERIALS AND METHODS

### Cells and reagents

Cell lines were obtained from ATCC with the exception of HKH2, kindly provided by Dr. Johannes Bos, Universitair Medisch Centrum, Utrecht and cultured as listed in Supplementary Table S1. Unless otherwise noted, all cells were grown and treated in uncoated tissue culture-treated flasks in a humidified atmosphere at 37°C and 5% CO_2_. Cell lines were pathogen-tested and genetically authenticated by short tandem-repeat analysis. Banked master stocks were returned to within approximately 6 months, or if inconsistencies in growth behavior were observed.

### Cell proliferation assays

Cells were seeded on poly-D-lysine coated plates (Corning) in their full culture media (Supplementary Table S1) and treated at 16–24 hrs post-plating for 72 hrs. Proliferation was assessed by CellTiter-Glo^®^ Luminescent Cell Viability Assay (Promega) according to manufacturer's instructions.

### Western blot procedure

Cells were treated with LY3009120 or DMSO as indicated and prepared for Western blotting as previously described [52]. Signal was detected by enhanced chemiluminescence and visualized on the ChemiDoc XRS instrument (Bio-Rad). Antibodies against ARAF (#4432), pBRAF S445 (#2696), pCRAF S338 (#9427), pEGFR Y1068 (#3777), EGFR (#4267), pRSK T359 (#8753), RSK1 (#9333), pMEK1/2 S217/S221 (#9154), pERK1/2 T202/Y204 (#4370), ERK1/2 (# 4696), pS6 S240/244 (“pS6”; #5364), rpS6 (“S6”; #2317), pAKT S473 (#4060), AKT (#2920), Rb (#9309), pMET Y1234/1235 (#3077), MET (#8198) (all from Cell Signaling Technology), BRAF (Santa Cruz Biotechnology, Inc. #sc-9002), CRAF (Bethyl Labs #A301-519A), pRb S780 (BD Biosciences #558385), and α-Tubulin (Sigma #T5168) were diluted in 5% BSA in 1x TBS-T.

### Cell cycle

Cells were treated with LY3009120 as indicated. Following treatment, cells were harvested, fixed with 70% ethanol and treated with 0.2 mg/mL DNase-free RNase (Sigma) and 0.02 mg/mL propidium iodide (PI) (Life Technologies) in 0.1% Triton X-100 (Sigma). PI intensity was measured by flow cytometry on a Beckman FC500 cytometer (Beckman Coulter, Inc.) and data were analyzed using ModFit (Verity Software).

### Immunofluorescence staining and high content imaging analysis

Cells were seeded on poly-D-lysine plates and were treated for the indicated times and concentrations of LY3009120 followed by fixation and staining as previously described [24]. Briefly, cells were fixed with 3.7% formaldehyde in PBS for 20 min and permeabilized with 0.1% Triton X-100 in PBS for 10 minutes at 25°C. Fixative was removed, cells were washed with PBS and blocked using 1% BSA in PBS for 1 hr at 25°C followed by incubation with primary antibodies against the proteins indicated for 1 hour at 25°C. Cells were washed with PBS and incubated with Hoechst nuclear stain and the secondary antibodies AlexaFluor-555 goat anti-mouse (Invitrogen) and Alexafluor-647 goat anti-rabbit (Invitrogen) for 1 hr at 25°C. Antibody and Hoechst staining were done in 1% BSA in PBS. Cells were washed with PBS and stored at 4°C until analysis. Cell images were captured on CellInsight™ CX5 High Content Screening Platform (Thermo Scientific) using the Target Activation algorithm at an image magnification of 10. Objects were identified using an algorithm to detect nuclear staining with Hoechst dye, and the relative levels of cleaved caspase 3 (Cell Signaling #9661), cleaved PARP (Cell Signaling #5625), pERK1/2 T202/Y204 (Cell Signaling #4370), pS6 S240/244 (Cell Signaling #5364), and p27 (Cell Signaling #3686) (all rabbit) and pHH3 (Cell Signaling #9706), S6 (Cell Signaling #2317), and ERK1/2 (Cell Signaling #4696) (all mouse) were determined through the intensities of Alexafluor-647 goat anti-rabbit (Invitrogen #A21245) and Alexafluor-555 goat anti-mouse (Invitrogen #A21425) respectively. Staining using the Click-iT^®^ TUNEL or Click-iT^®^ EdU kits (Life Technologies) was carried out according to manufacturer's instructions. A minimum of 2500 individual cellular images or 9 fields were captured for each condition.

### Gene expression analyses

Gene expression analysis was carried out using QuantiGene beads (Affymetrix) according to the manufacturer's instructions. Quantification of signal was obtained using MirrorBall (TTP Labtech).

### siRNA transfections

Transfections were performed using Lipofectamine RNAiMAX (Life Technologies) according to manufacturer's instructions. OnTarget Plus siRNA pools targeting *ARAF* (cat # L-003563-00), *BRAF* (cat # L-003460-00), *CRAF* (cat # L-003601-00) and non-targeting control (cat #D-001810-10) were obtained from Dharmacon (Thermo Scientific).

### *In vivo* xenograft models

5 million tumor cells in inoculation media (HBSS+Matrigel 1:1 mix) were implanted subcutaneously in the right hind flank of female NIH nude rats (Taconic Biosciences). When tumors reached ∼400 mm^3^, animals were randomized into groups of 8–10 and treated as indicated in the respective figure legends. LY3009120 was administered orally and animals were monitored for toxicity as previously described [15, 53]. Vehicle control was 20% Captisol^®^ for studies with Colo 205, HT-29 and HCT 116 and 1% HEC/0.25% Tween 80/0.05% Antifoam for the Colo 320HSR study. All procedures and techniques were performed in accordance with the institutional guidelines of the American Association for Laboratory Animal Care and monitored by the Eli Lilly and Company Animal Care and Use Committee.

### Tumor xenograft preparation for western blotting

Tumors harvested upon study completion were briefly thawed and 20-30 mg of tissue sample was placed in a tube containing Lysing Matrix A (MP Biomedicals) and 650 μL of lysis buffer (XY buffer: 1% Triton X100, 25 mM Tris pH7.5, 150mM NaCl, 1mM EDTA/1mM EGTA) supplemented with 3X Halt protease/phosphatase inhibitors (Thermo Scientific). Samples were homogenized for 20 seconds in a FastPrep FP120 Cell Disrupter (Thermo Electron) and allowed to sit on ice for 1 hr. The non-fatty layer of each sample was collected and spun at 3000rpm for 10 minutes at 4°C; supernatants were quantified for protein using the DC Protein Assay (Bio-Rad #500-0116) as previously described [52] and according to the instructions of the manufacturer.

### Generation of an LY3009120-resistant cell line

HCT 116 LY3009120-resistant cells (HCT 116 2000) were generated by continuous culture in 1 μM LY3009120 for 4-6 weeks until confluence was achieved. Resistant cells were further selected and maintained by culturing in 2 μM LY3009120 until confluence was achieved. Resistance to LY3009120 and other kinase inhibitors was evaluated by CellTiter Glo as described above.

### RAS activation assay

Examination of RAS-activation status was performed using the active RAS Pull-Down and Detection Kit (Thermo Scientific) according to manufacturer's instructions.

### Statistical analysis

Data from two biological replicates of the effects of siRNA knockdown on each of 3 cell lines were analyzed with a “variance components mixed effects” model with assay run as a random effect and the type of siRNA knockdown used as a fixed effect. Multiple comparisons were conducted using Tukey's HSD to adjust for multiplicity. All analyses were conducted using JMP v11.1.1 (SAS). The analyses of the CRC xenograft models were conducted with a mixed effects ANOVA model.

## SUPPLEMENTARY MATERIALS FIGURES AND TABLES


