# Supplementary material for: LY3009120, a panRAF inhibitor, has significant anti-tumor activity in BRAF and KRAS mutant preclinical models of colorectal cancer
Source: Oncotarget. 2016 Dec 16;8(6):9251–66. doi: 10.18632/oncotarget.14002 (PMC5354729; doi:10.18632/oncotarget.14002)
Supplement: Supplementary file 1 [file oncotarget-08-9251-s001.pdf]

LY3009120, a panRAF inhibitor, has significant anti-tumor activity in BRAF and KRAS mutant preclinical models of colorectal cancer

Supplementary Materials

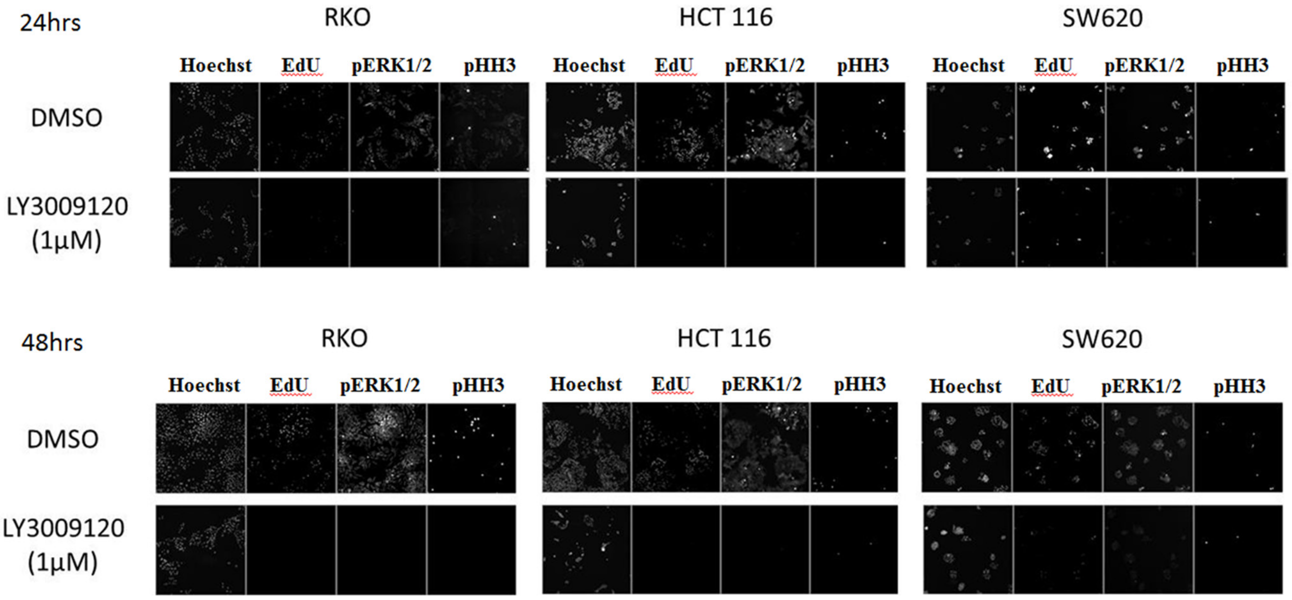

Supplementary Figure S1: ***BRAF***^{mut} **(RKO) or** ***KRAS***^{mut} **(HCT 116 and SW620) CRC cell lines were treated with 1 µM LY3009120 or DMSO control, fixed at 24 or 48 hrs and stained for immunofluorescence with Hoechst stain (nuclei), Click-iT® EdU and immunofluorescent antibodies against pERK1/2 T202/Y204 and pHH3 S10 as indicated.**

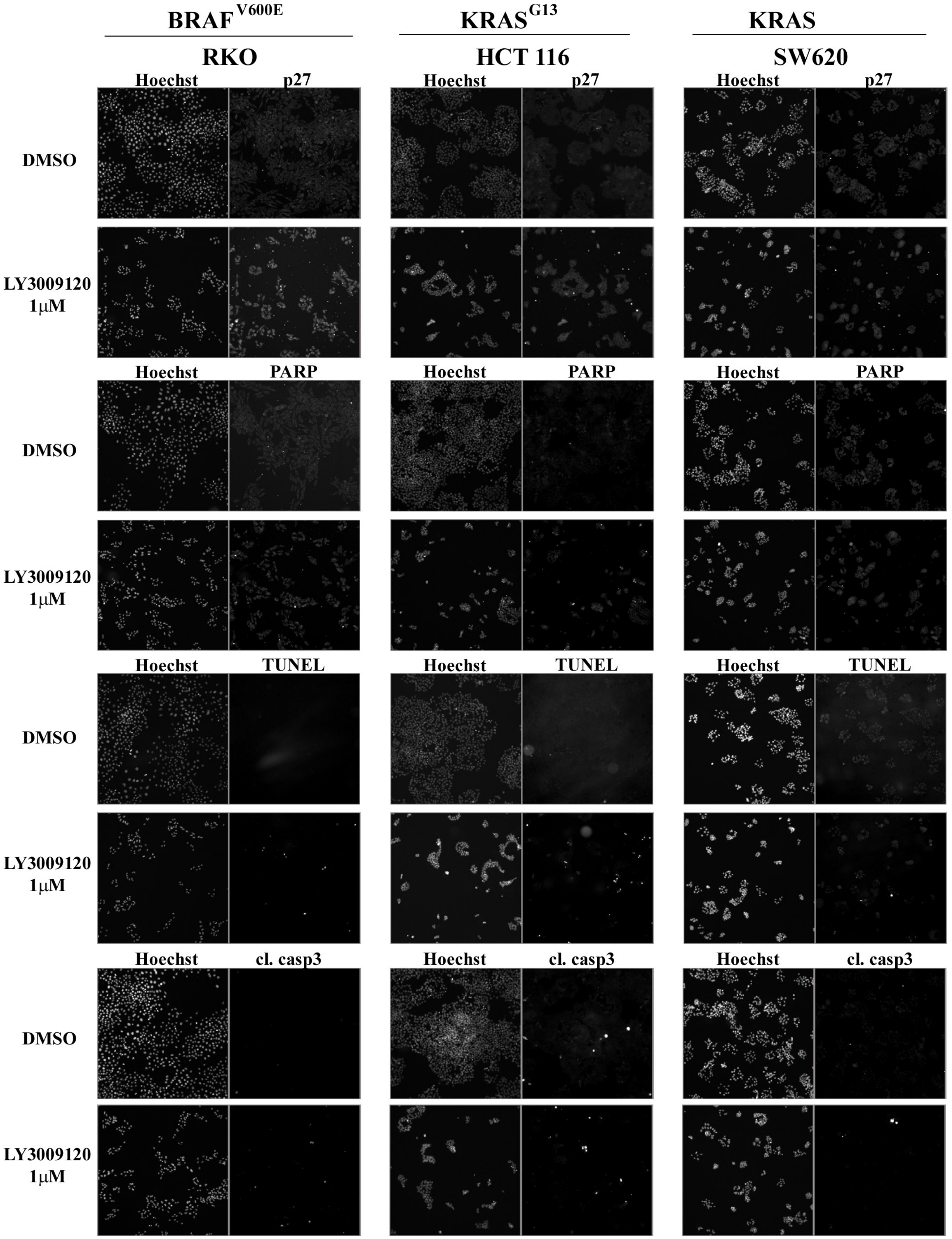

Supplementary Figure S2: Cells were treated with 1 μM LY3009120 or DMSO control and fixed at 48 hrs post-treatment. Cells were stained for immunofluorescence analysis with Hoechst stain and antibodies against the proteins indicated.

Supplementary Table S1: Cell culture media of the CRC cell lines used

Cell line	Cell Culture Medium
Colo 205	RPMI + 10% FBS
HT-29	RPMI + 10% FBS
RKO	DMEM + 10% FBS
HCT-15	DMEM + 10% FBS
DLD-1	RPMI + 10% FBS
HCT 116	McCoy's + 10% FBS
HKH2	McCoy's + 10% FBS
LoVo	MEM + 10% FBS
SW480	DMEM + 10% FBS
SW620	DMEM + 10% FBS
SW837	DMEM + 10% FBS
Colo 320HSR	RPMI + 10% FBS
SNU-C1	RPMI + 10% FBS
NCI-H716	RPMI + 10% FBS
SW48	DMEM + 10% FBS

Supplementary Table S2: Statistical analysis of the effects of siRNA-mediated knockdown on the proliferation of HCT 116 cells

Cell Line	siRNA Knock Down	vs. siRNA Knock Down	Difference	*p*-Value
HCT 116	ctrl	A	18.50425	0.1545
HCT 116	ctrl	AB	16.87784	0.2488
HCT 116	ctrl	AC	29.2205	0.0017
HCT 116	ctrl	BC	33.50357	0.0002
HCT 116	ctrl	ABC	64.74037	<.0001
HCT 116	A	AC	10.71625	0.7883
HCT 116	A	BC	14.99932	0.396
HCT 116	A	ABC	46.23612	<.0001
HCT 116	B	ctrl	18.27666	0.1658
HCT 116	B	A	36.78091	<.0001
HCT 116	B	C	1.43862	1
HCT 116	B	AB	35.1545	<.0001
HCT 116	B	AC	47.49717	<.0001
HCT 116	B	BC	51.78023	<.0001
HCT 116	B	ABC	83.01703	<.0001
HCT 116	C	ctrl	16.83804	0.2515
HCT 116	C	A	35.34229	<.0001
HCT 116	C	AB	33.71588	0.0002
HCT 116	C	AC	46.05854	<.0001
HCT 116	C	BC	50.34161	<.0001
HCT 116	C	ABC	81.57841	<.0001
HCT 116	AB	A	1.62641	1
HCT 116	AB	AC	12.34266	0.6456
HCT 116	AB	BC	16.62573	0.2662
HCT 116	AB	ABC	47.86253	<.0001
HCT 116	AC	BC	4.28307	0.9986
HCT 116	AC	ABC	35.51987	<.0001
HCT 116	BC	ABC	31.2368	0.0006

Supplementary Table S3: Statistical analysis of the effects of siRNA-mediated knockdown on the proliferation of SW620 cells

Cell Line	siRNA Knock Down	vs. siRNA Knock Down	Difference	*p*-Value
SW620	ctrl	A	0.66995	1
SW620	ctrl	AB	11.32169	0.3612
SW620	ctrl	AC	14.01361	0.1291
SW620	ctrl	BC	18.04881	0.0157
SW620	ctrl	ABC	48.02203	<.0001
SW620	A	AB	10.65174	0.4416
SW620	A	AC	13.34366	0.1721
SW620	A	BC	17.37887	0.0232
SW620	A	ABC	47.35208	<.0001
SW620	B	ctrl	8.66138	0.6973
SW620	B	A	9.33132	0.6122
SW620	B	AB	19.98307	0.0047
SW620	B	AC	22.67498	0.0008
SW620	B	BC	26.71019	<.0001
SW620	B	ABC	56.6834	<.0001
SW620	C	ctrl	11.58822	0.3314
SW620	C	A	12.25817	0.2626
SW620	C	B	2.92685	0.9991
SW620	C	AB	22.90991	0.0006
SW620	C	AC	25.60183	<.0001
SW620	C	BC	29.63704	<.0001
SW620	C	ABC	59.61025	<.0001
SW620	AB	AC	2.69192	0.9995
SW620	AB	BC	6.72712	0.8935
SW620	AB	ABC	36.70034	<.0001
SW620	AC	BC	4.03521	0.9935
SW620	AC	ABC	34.00842	<.0001
SW620	BC	ABC	29.97321	<.0001

Supplementary Table S4: Statistical analysis of the effects of siRNA-mediated knockdown on the proliferation of RKO cells

Cell Line	siRNA Knock Down	vs. siRNA Knock Down	Difference	*p*-Value
RKO	ctrl	A	11.04178	0.5907
RKO	ctrl	B	30.9041	<.0001
RKO	ctrl	AB	45.82386	<.0001
RKO	ctrl	AC	12.50319	0.429
RKO	ctrl	BC	27.00062	0.0005
RKO	ctrl	ABC	49.94463	<.0001
RKO	A	B	19.86233	0.0274
RKO	A	AB	34.78208	<.0001
RKO	A	AC	1.46142	1
RKO	A	BC	15.95884	0.1465
RKO	A	ABC	38.90285	<.0001
RKO	B	AB	14.91975	0.2116
RKO	B	ABC	19.04053	0.0404
RKO	C	ctrl	0.48766	1
RKO	C	A	11.52944	0.5359
RKO	C	B	31.39176	<.0001
RKO	C	AB	46.31152	<.0001
RKO	C	AC	12.99085	0.3786
RKO	C	BC	27.48828	0.0004
RKO	C	ABC	50.43229	<.0001
RKO	AB	ABC	4.12077	0.9971
RKO	AC	B	18.40091	0.054
RKO	AC	AB	33.32066	<.0001
RKO	AC	BC	14.49743	0.2431
RKO	AC	ABC	37.44144	<.0001
RKO	BC	B	3.90348	0.9979
RKO	BC	AB	18.82324	0.0447
RKO	BC	ABC	22.94401	0.0055
